# Spatial Differentiation and Community Structure Characteristics of Soil Microorganisms at Variable Hyphosphere Distances in Forest Cultivation Systems of *Morchella*

**DOI:** 10.3390/microorganisms14051003

**Published:** 2026-04-29

**Authors:** Yan Zhang, Yingfei Xu, Bin Peng, Xun Li, Hongliang Ma

**Affiliations:** Key Laboratory of Ecological Protection and Characteristic Industry Cultivation in Hengduan Mountain Area of Sichuan Education Department and Ganzi Prefecture, Sichuan Minzu College, Kangding 626001, China; zhangyanyb@163.com (Y.Z.); xuyf20036@163.com (Y.X.); 200003@scun.edu.cn (B.P.); mhliang2019@163.com (H.M.)

**Keywords:** *Morchella*, understory cultivation, hyphosphere soil, microbial community

## Abstract

Under-forest cultivation of morels is increasingly constrained by soil ecological deterioration, which has become a major obstacle to its sustainable development. This study characterized hyphosphere soil microbiomes of *Morchella sextelata* M. Kuo under pine canopy at four distances from the fruiting body: 0 cm (R), 20 cm (R_20_), 40 cm (R_40_), and uncultivated control (CK). Bacterial and fungal community composition and diversity were analyzed using Illumina NovaSeq high-throughput sequencing. Results showed that the dominant bacterial phyla were Proteobacteria and Acidobacteriota, with *RB41*, *Sphingomonas*, and *Dongia* as the dominant genera. Relative to CK, the abundances of Acidobacteriota and *RB41* in R increased by 4.45% and 6.16%, respectively, whereas R_20_ was enriched in Proteobacteria (+7.77%), *Sphingomonas* (+0.95%), *Dongia*, and *Bradyrhizobium*. For fungi, Ascomycota and Basidiomycota were the dominant phyla, with the principal genera being *Sebacina*, Microbotryales_gen_Incertae_sedis, and *Oidiodendron*. Compared with CK, morel cultivation decreased the abundances of Ascomycota and *Oidiodendron*, with the greatest reductions in R_20_ (by 8.73% and 3.67%, respectively), while increasing the abundances of Basidiomycota, *Sebacina*, and Microbotryales_gen_Incertae_sedis, again most markedly in R_20_, by 17.56%, 14.82%, and 5.74%, respectively. Morel cultivation significantly reduced microbial diversity and evenness (Shannon, Simpson, and Pielou), with the lowest diversity and highest dominance in Zone R. Partial least squares structural equation modeling (PLS-SEM) revealed that soil chemical properties and enzyme activities negatively drove dominant bacterial genera but positively drove dominant fungal genera. Overall, under-forest cultivation of *M. sextelata* significantly reduced hyphosphere microbial diversity and reshaped microbial community structure in a distance-dependent manner: Zone R was dominated by Acidobacteriota; Zone R_20_ was enriched with nitrogen-cycling beneficial bacteria (*Dongia*, *Sphingomonas*, and *Bradyrhizobium*) and beneficial fungi (*Sebacina* and Microbotryales_gen_Incertae_sedis); Zone R_40_ exhibited relatively optimal fungal diversity.

## 1. Introduction

Morels are rare edible mushrooms of high economic value and are highly prized for their distinctive flavor as well as their exceptional nutritional and medicinal properties. As such, they have become a key component of China’s rapidly expanding emerging edible mushroom industry, and global production of edible mushrooms has exceeded 40 million tons annually [1,2]. To date, 28 species of the genus *Morchella* have been identified worldwide, of which only eight have been reported in China. Among them, the currently dominant cultivated species—*Morchella sextelata*, *Morchella eximia*, and *Morchella importuna*—display several agronomically desirable traits, including strong environmental adaptability, short cultivation cycles, relatively simple management requirements, high yields, and superior temperature tolerance of young fruiting bodies [3,4]. However, achieving sustainable cultivation of morels remains an urgent challenge.

During growth, morels selectively absorb soil nutrients, for example causing a 15–30% reduction in available phosphorus [5], while also altering soil pH, decreasing total nitrogen and organic matter contents, and potentially enriching potassium [5,6]. More importantly, with increasing years of continuous cultivation, the abundance and diversity of soil bacterial communities decline significantly, and the depletion of beneficial microbial taxa is considered a major driver of continuous-cropping obstacles [7,8]. Therefore, morel cultivation can profoundly reshape soil physicochemical properties and microbial community composition [6,9], thereby affecting normal fructification.

Microorganisms inhabiting the soil adjacent to roots rely on root-secreted compounds, exudates, and sloughed cells for growth. They are essential for maintaining soil health and play critical roles in both environmental and ecological soil functions [10,11]. As a soil-borne fungus, morel develops a dense hyphal network that creates a unique hyphosphere-like microenvironment [12]. The microbial communities within this zone are closely associated with morel growth and development. Previous studies have shown that the abundance of bacteria, actinomycetes, and specific fungal taxa is significantly higher in soils from morel-growing areas than in non-growing areas [13]. Morel growth, particularly the development of the subterranean mycelial network and the frost-like mycelial mats it forms [12], strongly drives nutrient dynamics and microbial metabolic activity in the surrounding soil [14], generating a complex “hyphae–nutrient–microbe” interaction network. For example, organic matter, alkali-hydrolyzable nitrogen, and available potassium reach their highest levels in the hyphosphere, whereas the peak in available phosphorus may occur about 50 cm away from where the morels grow [15]. Morels also engage in close interactions with specific microorganisms: hyphal secretions recruit and depend on particular bacterial colonizers that provide nutrients, while fungal metabolites such as oxalic acid, together with secreted enzymes including cellulase and amylase, markedly influence microbial community structure and function, thereby regulating soil enzyme activities and nutrient cycling efficiency [16]. Elucidating the spatiotemporal distribution patterns of morel-associated hyphosphere microorganisms is therefore of considerable importance for optimizing cultivation practices and promoting the sustainable development of the industry.

Under-forest semi-natural cultivation, which makes efficient use of forestland resources, alleviates pressure on arable land, protects wild morel populations, and improves economic returns, represents an important direction for the green and sustainable development of the morel industry. Among cultivated species, *M. sextelata* is the most widely grown and has shown particular potential for improving soil structure and enhancing organic matter accumulation because of its well-developed mycelial network and strong lignocellulose-degrading capacity, as reflected by its high carboxymethyl cellulase and amylase activities [17,18]. However, the spatial gradient patterns of morel hyphosphere microbial communities and their responses to increasing distance from the hyphosphere remain poorly understood. We hypothesized that the mycelial network of *Morchella sextelata* may shape spatial gradients in soil physicochemical properties and nutrient availability, thereby exerting distance-dependent filtering on soil microbial communities. In this study, soil samples were collected at different distances from the morel hyphosphere and analyzed using high-throughput microbial sequencing to characterize the structural and diversity changes of soil microbial communities along a hyphosphere-distance gradient. The findings of this study will provide an important theoretical basis for a deeper understanding of the microecology of the morel hyphosphere and for the optimization of cultivation systems, particularly under-forest semi-natural cultivation, thereby supporting the healthy and sustainable development of the morel industry.

## 2. Materials and Methods

### 2.1. Study Site

The study site was located in Zhuangshang Village, Guzan Town, Kangding City, Sichuan Province, China (102°12′36″ E, 29°58′48″ N), at an elevation of 1850 m. The region is characterized by a plateau mountain climate. The mean annual temperature is 12.5 °C, the mean annual frost-free period is 210 days, the mean annual precipitation is 850 mm, and the mean annual relative air humidity is 65%, with distinct wet and dry seasons. The soil in this region is brown sandy forest soil. The dominant tree species is *Pinus densata*, with shrubs such as *Rhododendron lapponicum* and a small number of *Viburnum acerifolium* and *Solidago decurrens*.

### 2.2. Experimental Design and Sample Collection

In October 2024, an experimental plot was established in a pine forest understory in Zhuangshang Village, Guzan Town, Kangding City, Sichuan Province, in an area with no previous history of edible mushroom cultivation. The plot area was 10 m^2^. The soil within the plot was prepared by land leveling, furrow opening, and ridge formation in November of the same year. *Morchella sextelata* spawn, which had been pre-cultured in the laboratory using wheat grains as the substrate, was then evenly broadcast onto the prepared soil surface and subsequently covered with soil.

Based on the microscopic evidence presented by Shi Jiansen et al. [19] indicating that *Morchella* primordia form in the topsoil, we collected soil samples from the 0–10 cm layer in April 2025 (the fruiting season). All sampling tools were sterilized with 75% ethanol prior to use. Sampling points were established at four locations: soil collected directly beneath the stem of the morel fruiting body (0 cm; R), as well as soil at distances of 20 cm (R_20_) and 40 cm (R_40_) from the fruiting body. A control group (CK) was also established in an uncultivated area at least 20 cm away from R_40_. Six sampling points were set in each area. At each sampling point, five subsamples were collected using a five-point sampling method and thoroughly mixed to form one composite soil sample of approximately 500 g. Each composite sample was placed in a polyethylene bag, properly labeled, and transported for further analysis. In total, 24 soil samples were collected across the four areas (4 distances × 6 replicates).

Each soil sample was then evenly divided into three portions. One portion of fresh soil was refrigerated and transported to the laboratory as soon as possible; after removal of stones, plant root residues, and other debris, it was used for soil microbial analysis. A second portion was transported to the laboratory, cleared of stones, root residues, and other impurities, immediately transferred into centrifuge tubes, and used for soil enzyme activity assays. The third portion was transported to the laboratory, air-dried, ground, and sieved for the determination of soil chemical properties, including pH, electrical conductivity (EC), soil organic carbon (SOC), total nitrogen (TN), and total phosphorus (TP).

### 2.3. Sample Analyses

#### 2.3.1. Determination of Soil Chemical Properties

Basic soil chemical properties were determined using standard methods. Soil pH and electrical conductivity (EC) were measured by the potentiometric method after mixing soil and water at ratios of 1:2.5 and 1:5, respectively. Soil total nitrogen was determined using the Kjeldahl method. Soil organic carbon was measured using an elemental analyzer (Elementar Analysensysteme GmbH, Langenselbold, Germany) [20]. Total phosphorus was determined by the acid dissolution–molybdenum antimony anti-colorimetric method.

The β-1,4-glucosidase (BG) and cellobiohydrolase (CBH) activities were determined using a fluorescent assay [21]. One gram of fresh soil sample was weighed into a 250 mL shaking flask, and 125 mL of sodium acetate buffer (50 mmol/L) was added, and the mixture was shaken for 1 h to prepare a soil suspension. In a 96-well microplate, 200 μL of the soil suspension and 50 μL of substrate solution (200 μmol/L) were added, and the plate was incubated in the dark at 20 °C for 4 h. Then 10 μL of NaOH solution (1 mol/L) was added. After standing for 1 min, the fluorescence intensity was measured using a microplate reader at an excitation wavelength of 365 nm and an emission wavelength of 450 nm. Urease (URE) activity was determined using the indophenol blue colorimetric method. Urease catalyzes the hydrolysis of urea to produce ammonia, which reacts with a color developer to form a blue complex. The content of ammonium nitrogen was determined spectrophotometrically and converted to urease activity. Catalase (CAT) activity was determined using a spectrophotometric method, with enzyme activity calculated based on the rate of absorbance decay of H_2_O_2_ at a wavelength of 240 nm, reflecting the soil’s ability to decompose hydrogen peroxide. Enzyme activity is calculated using the method described by DeForest [22], with units of nmol/(g·h).

#### 2.3.2. Analysis of Soil Microbial Communities

Soil microbial communities were analyzed by high-throughput sequencing conducted by Novogene Co., Ltd., Beijing, China. Briefly, PCR amplification was first performed, followed by library construction and paired-end sequencing on the Illumina NovaSeq platform (Beijing, China). The primers and ITS sequences used to determine microbial diversity are shown in Table 1. After read merging and quality filtering, operational taxonomic units (OTUs) were clustered or amplicon sequence variants (ASVs) were denoised. The resulting high-quality sequences were then subjected to taxonomic annotation and abundance analysis to characterize the species composition of each sample. All α-diversity indices (such as Chao1, Pielou, Shannon, dominance, and Simpson) were derived from sequencing data generated by the Novogene Co., Subsequent analyses of α-diversity and β-diversity were performed to evaluate microbial diversity patterns among samples. Amplified PCR products were further extracted and purified, and sequencing data were processed and analyzed using the QIIME2 platform. The resulting datasets were used to compare differences in microbial community composition among treatments. DNA sample purification (RNA digestion) conditions: Take 3 μL of the original solution + 6 μL of ddH_2_O + 1 μL of RNase A (original concentration 10 mg/mL); incubate at 37 °C for 15 min. Take 2 μL for electrophoresis. Add 15 µL of Phusion ^®^ High-Fidelity PCR Master Mix (New England Biolabs), 0.2 µM of primers, and 10 ng of genomic DNA template to all PCR mixtures. Perform an initial denaturation at 98 °C for 1 min, followed by 30 cycles at 98 °C (10 s), 50 °C (30 s), and 72 °C (30 s), followed by a final extension at 72 °C for 5 min.

### 2.4. Data Processing and Statistical Analysis

One-way analysis of variance (ANOVA) was performed in IBM SPSS Statistics 27 to analyze bacterial and fungal alpha diversity as well as the relative abundance of dominant phyla. Duncan’s multiple range test was used to assess the significance of differences among treatments, with *p* < 0.05 considered statistically significant. Figures related to bacterial and fungal communities were generated using Origin 2024. Principal coordinates analysis (PCoA) was performed, and structural equation models describing the relationships among dominant bacterial and fungal genera and microbial diversity were constructed in R 4.5.1 using the lavaan, ggplot2, and semPlot packages.

## 3. Results

### 3.1. Amplicon Sequence Characteristics of Soil Bacterial and Fungal Communities

**Figure 1:**
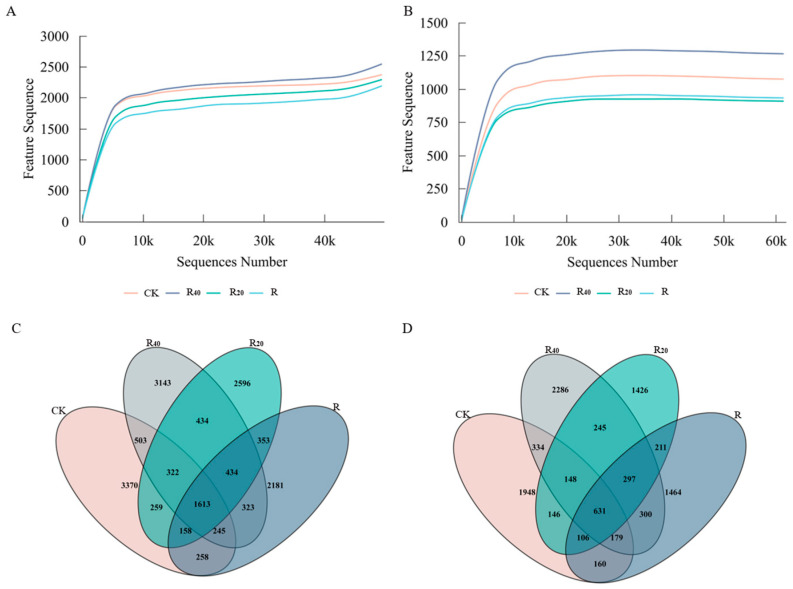
Amplification characteristic sequences of soil bacterial and fungal communities. (**A**): bacterial rarefaction curve; (**B**): fungal rarefaction curve; (**C**): bacterial Venn diagram; (**D**): fungal Venn diagram.

Amplicon sequence variants data from the different bacterial and fungal groups were used to evaluate sequencing saturation. For all treatments (CK, R, R_20_, and R_40_), the rarefaction curves increased rapidly at low sequencing depth and then gradually approached a plateau as sequencing depth increased, with stabilization beginning at approximately 10,000 amplicon sequence variants, indicating that the sequencing depth was sufficient to capture the majority of microbial diversity present in the samples. The rarefaction curves for R_40_ and CK consistently remained above those for R_20_ and R, suggesting that microbial alpha diversity may have been lower in R_20_ and R (Figure 1A,B).

A total of 1613 bacterial amplicon sequence variants were shared among all four groups, whereas the numbers of unique bacterial amplicon sequence variants in R, R_20_, R_40_, and CK were 2181, 2596, 3143, and 3370, respectively. Thus, the numbers of treatment-specific bacterial amplicon sequence variants were approximately 1.5–2 times greater than the number of shared amplicon sequence variants (Figure 1C). For fungi, 631 amplicon sequence variants were shared among all four treatments, whereas the numbers of unique fungal amplicon sequence variants in R, R_20_, R_40_, and CK were 1464, 1426, 2286, and 1948, respectively. The numbers of unique fungal amplicon sequence variants were therefore approximately two- to threefold greater than the number of shared amplicon sequence variants (Figure 1D).

### 3.2. Compositional Characteristics of Soil Microbial Communities at Different Distances from the Hyphosphere Under Under-Forest Morel Cultivation

**Figure 2:**
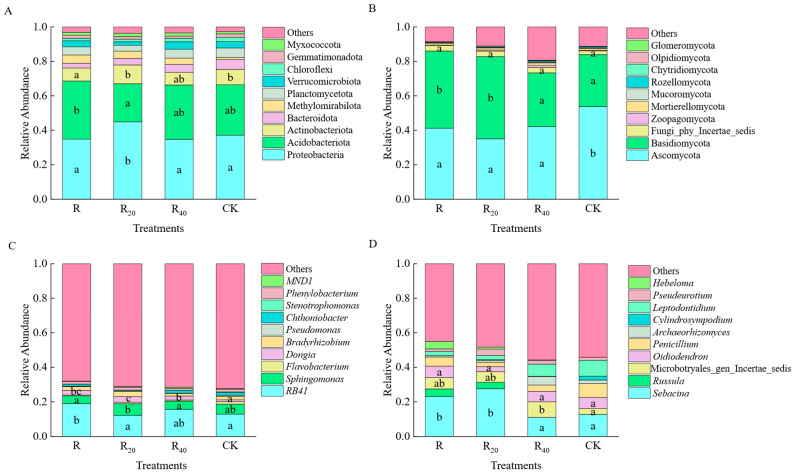
Bar chart of species abundance for bacteria and fungi under different treatments. (**A**): bacterial phyla; (**B**): fungal phyla; (**C**): bacterial genera; (**D**): fungal genera. Different lowercase letters in the figure indicate significant differences in the same bacterial and fungi phylum among different groups (the same as below).

The top 10 most abundant bacterial and fungal phyla at different distances from the hyphosphere are shown in Figure 2A,B. The dominant bacterial phyla were broadly similar across all sampling distances. Proteobacteria was the most abundant bacterial phylum (34.77–44.94%), followed by Acidobacteriota (22.09–33.72%). Compared with CK, R_20_ significantly increased the relative abundances of Proteobacteria (+7.77%) and Actinobacteriota (+1.99%) (*p* < 0.05). In contrast, R significantly increased the abundance of Acidobacteriota (+4.45%) (*p* < 0.05), whereas R_20_ decreased it (−7.18%). The relative abundance of Bacteroidota decreased in R, R_20_, and R_40_ compared with CK. The relative abundances of Proteobacteria and Actinobacteriota followed the pattern R_20_ > R > R_40_, whereas Acidobacteriota followed the pattern R > R_40_ > R_20_ (Figure 2A).

The dominant fungal phyla were Ascomycota (35.06–53.79%) and Basidiomycota (30.15–47.71%). Compared with CK, morel cultivation significantly reduced the relative abundance of Ascomycota, with the greatest decrease observed in R_20_ (18.73%) (*p* < 0.05), while significantly increasing the relative abundance of Basidiomycota, again with the largest increase in R_20_ (17.56%) (*p* < 0.05). CK had the highest relative abundance of Ascomycota and the lowest relative abundance of Basidiomycota (Figure 2B).

The top 10 most abundant bacterial and fungal genera are shown in Figure 2C,D. *RB41* was the most abundant bacterial genus-level taxon (12.11–18.97%), followed by *Sphingomonas* (4.50–6.77%). Compared with CK, the relative abundance of *RB41* increased significantly in R and R_40_ (by 6.16% and 2.82%, respectively) (*p* < 0.05), but decreased slightly in R_20_ (−0.70%). By contrast, the relative abundance of *Sphingomonas* decreased in R and R_40_ (by 1.32% and 1.09%, respectively), but increased in R_20_ (+0.95%). The relative abundances of *Dongia* and *Bradyrhizobium* were both below 4%, but both reached their highest values in R_20_ (Figure 2C).

The three most dominant fungal genera were *Sebacina*, Microbotryales_gen_Incertae_sedis, and *Oidiodendron*. Compared with CK, the relative abundance of *Sebacina* increased significantly in both R and R_20_, with the largest increase observed in R_20_ (+14.82%) (*p* < 0.05). Microbotryales_gen_Incertae_sedis increased significantly in R, R_20_, and R_40_, with the greatest increase in R_40_ (+5.73%) (*p* < 0.05). By contrast, *Oidiodendron* decreased significantly in R_20_ (−3.67%) (*p* < 0.05). The relative abundance of *Leptodontidium* was lower in all cultivation treatments than in CK (Figure 2D).

**Figure 3:**
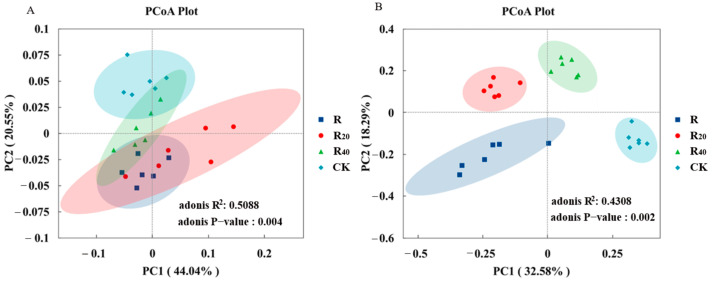
PCoA analysis of different treatments on bacteria and fungi. (**A**): bacteria; (**B**): fungi.

Principal coordinates analysis (PCoA) based on weighted UniFrac distances was used to assess differences in soil microbial community structure. For bacteria, PC1 and PC2 explained 44.04% and 20.55% of the total variation, respectively, together accounting for 64.59% of the observed community variation (Figure 3A). For fungi, the first two axes jointly explained 50.87% of the total variation, with PC1 and PC2 accounting for 32.58% and 18.29%, respectively (Figure 3B). Adonis analysis further showed that hyphosphere distance had a highly significant effect on both bacterial and fungal community structure (*R*^2^ = 0.5088, *p* < 0.01; *R*^2^ = 0.4308, *p* < 0.01). For bacteria, R and R_20_ were clearly separated from CK, indicating that their bacterial community compositions differed markedly from that of the control; R and R_20_ clustered relatively closely, whereas R_40_ partially overlapped with CK, suggesting a smaller difference between these two groups. For fungi, all four treatments were clearly separated, indicating significant differences in fungal community composition among all groups. Overall, morel cultivation significantly altered both bacterial and fungal community composition, and the magnitude of change in bacterial composition increased with proximity to the mycelial zone.

### 3.3. Microbial Diversity in Hyphosphere Soils at Different Distances Under Under-Forest Morel Cultivation

The alpha-diversity indices of soil bacteria and fungi differed significantly among hyphosphere distances (*p* < 0.05) (Table 2). For soil bacteria, the Pielou, Shannon, and Simpson indices were all significantly highest in CK, followed by R_40_ and R_20_, and were significantly lowest in R. The Dominance index showed the opposite trend (*p* < 0.05). No significant difference was observed in the bacterial Chao1 index (*p* > 0.05), although the values followed the order R_40_ > CK > R_20_ > R.

For soil fungi, the Chao1 index was significantly highest in R_40_, followed by CK and R, and was significantly lowest in R_20_ (*p* < 0.05). The Pielou, Shannon, and Simpson indices were all significantly highest in R_40_, intermediate in CK and R_20_, and lowest in R, whereas the Dominance index displayed the opposite pattern (*p* < 0.05). Taken together, morel cultivation reduced both bacterial diversity (with the smallest reduction in R_40_) and fungal diversity (except in R_40_), while increasing community dominance, with the highest Dominance values observed in R.

### 3.4. Correlations of Soil Chemical Properties with Bacterial and Fungal Communities and Diversity

#### 3.4.1. Mantel Tests Linking Soil Chemical Properties to Bacterial and Fungal Community Composition and Diversity

**Figure 4:**
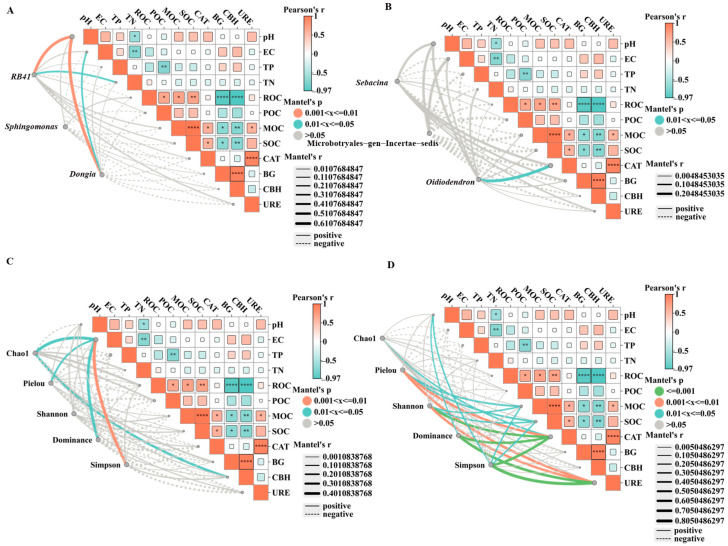
Mantel test of soil chemical properties with dominant bacterial and fungal genera and diversity. (**A**): R; (**B**): R_20_; (**C**): R_40_; (**D**): CK. * indicates a significant correlation at the *p* < 0.05 level; ** indicates a highly significant correlation at the *p* < 0.01 level; **** indicates an extremely significant correlation at the *p* < 0.001 level.

Mantel tests were used to assess the relationships between soil chemical properties and the dominant bacterial and fungal genera. For bacteria, soil pH was very significantly positively correlated with *RB41* and *Dongia* (*p* < 0.01), while total nitrogen (TN) was significantly positively correlated with *RB41*, and electrical conductivity (EC) was significantly positively correlated with *Dongia* (*p* < 0.05) (Figure 4A). For fungi, catalase (CAT) activity was significantly positively correlated with *Oidiodendron* (*p* < 0.05) (Figure 4B).

Mantel tests were also performed to evaluate relationships between soil chemical properties and bacterial and fungal alpha diversity. For bacteria, EC was very significantly positively correlated with the Simpson index, and significantly positively correlated with the Chao1, Pielou, and Dominance indices, while cellobiohydrolase (CBH) activity was significantly positively correlated with Chao1 (Figure 4C). For fungi, CAT and urease (URE) were very significantly positively correlated with the Pielou, Shannon, Dominance, and Simpson indices. CAT was also significantly positively correlated with Simpson and Chao1. Mineral-associated organic carbon (MOC) and soil organic carbon (SOC) were significantly positively correlated with Shannon, Dominance, and Simpson, while MOC was significantly positively correlated with Chao1 and SOC was significantly positively correlated with Pielou. In addition, pH was significantly positively correlated with Dominance and Simpson (Figure 4D).

#### 3.4.2. Structural Equation Modeling of Dominant Bacterial and Fungal Genera, Diversity, and Soil Chemical Properties

**Figure 5:**
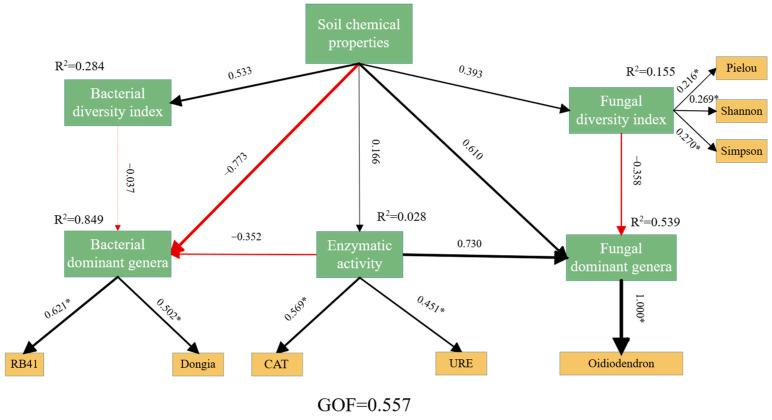
Structural equation model of soil chemical properties and soil microorganisms. The R^2^ values in the figures represent the proportion of variance, and GOF indicates the goodness of fit. Red lines represent statistically significant negative influences, while bold black lines represent statistically significant positive influences. The values on the arrows indicate the path coefficients. The thicker the lines and arrows, the stronger the correlation. * indicates that the load coefficient is significant at the 0.05 level.

Partial least-squares path modeling was used to analyze the direct and indirect effects of soil chemical properties for soil microorganisms. GOF is used to assess the goodness of fit of a model, a GOF value greater than 0.5 indicates a good fit. The results showed the GOF was 0.557, supporting a good model fit for the subsequent path analysis (Figure 5). Soil chemical properties were key environmental factors driving microbial community structure, but their effects on dominant bacterial and fungal genera were diametrically opposed: they significantly and negatively drive dominant bacterial genera (with path coefficients of −0.773, *p* < 0.05), while positively influencing dominant fungal genera (with path coefficients of 0.610, *p* < 0.05), explaining 84.9% and 53.9% of the variance in these two groups, respectively. Soil chemical properties had a weak positive effect on bacterial and fungal diversity (with path coefficients of 0.533 and 0.393, respectively). Enzyme activity, as an important functional mediator independent of soil chemical properties, significantly positively regulates dominant fungal genera (with path coefficients of 0.730, *p* < 0.05) but negatively influences dominant bacterial genera (with path coefficients of −0.352, *p* < 0.05), with CAT and URE serving as key indicators. Furthermore, bacterial diversity had a negative effect on dominant genera, whereas fungal diversity was significantly negatively correlated with dominant fungal genera. In summary, soil chemical properties and enzyme activity jointly shaped the differentiated ecological adaptation strategies of bacteria and fungi.

### 3.5. Co-Occurrence Network Patterns of Soil Bacteria and Fungi

**Figure 6:**
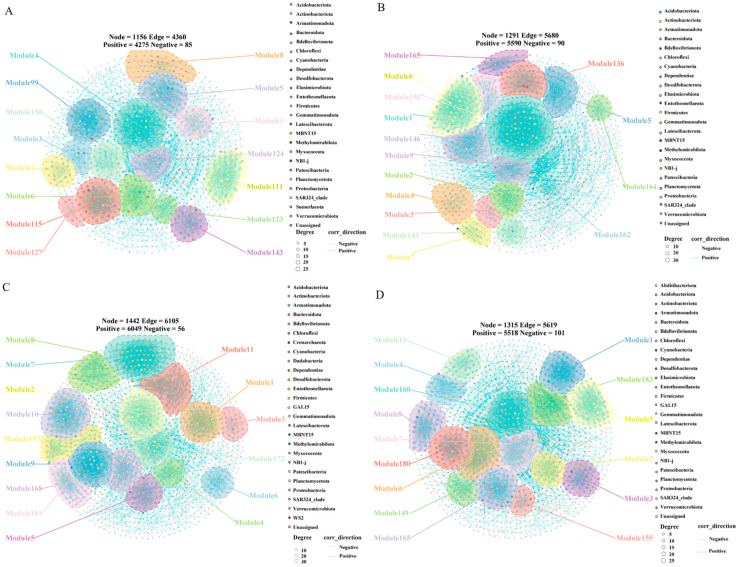
Bacterial co-occurrence network analysis. (**A**): R; (**B**): R_20_; (**C**): R_40_; (**D**): CK.

**Figure 7:**
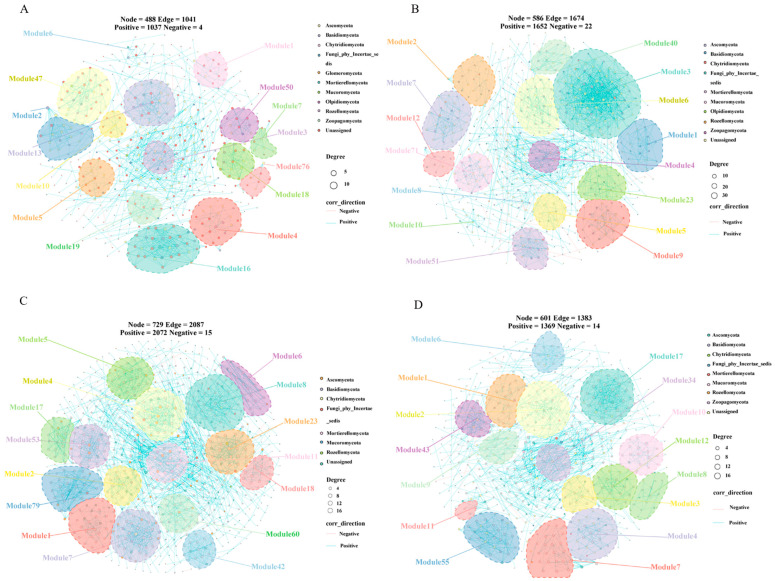
Fungal co-occurrence network analysis. (**A**): R; (**B**): R_20_; (**C**): R_40_; (**D**): CK.

The visualized co-occurrence networks of bacterial and fungal communities under different treatments are shown in Figure 6 and Figure 7, respectively, and were used to illustrate node connectivity and overall network structure. In these networks, different colored regions represent distinct modules, indicating groups of nodes with tighter internal connections, and the labels on the right indicate their taxonomic attributes. Each network node represents a bacterial or fungal community taxon, and node colors correspond to different modules. The modules are neither sequentially numbered nor continuously arranged because taxa were filtered by retaining only those present in at least two samples and with sequence abundance ≥3%.

The bacterial taxa included in the co-occurrence networks (Figure 6A–D) were derived mainly from Proteobacteria, Acidobacteriota, and Actinobacteriota. Across soils at different hyphosphere distances, the bacterial network in R_40_ showed the highest number of nodes and edges, the greatest number of positive associations, the highest complexity and stability, and the highest ratio of positive to negative edges (110.11), followed by R_20_ and CK, whereas R showed the lowest network complexity (Figure 6, Table 3).

Similarly, the fungal taxa included in the co-occurrence networks (Figure 7A–D) were derived mainly from Ascomycota and Basidiomycota. Among the different hyphosphere-distance soils, the fungal network in R_40_ also exhibited the highest numbers of nodes and edges, the greatest number of positive associations, and the highest complexity and stability, followed by R_20_ and CK, whereas R had the simplest and least stable network structure but had the highest ratio of positive to negative edges (249.00) (Figure 7, Table 4).

Overall, the bacterial community network was more complex than the fungal network. Moreover, microbial interactions and connectivity were more intricate and tighter in soils located farther from the hyphosphere than in soils immediately adjacent to the hyphosphere. Differences in the ratio of positive to negative edges reveal that bacterial interactions are dominated by a balance of competition and cooperation, while fungal interactions are characterized by high levels of cooperation, forming a symbiotic system with morels. These patterns are consistent with the results of the species composition and community diversity analyses described above.

## 4. Discussion

### 4.1. Effects of Under-Forest Morel Cultivation on Bacterial Community Composition and Diversity in Hyphosphere Soils at Different Distances

The structure of soil microbial communities is regulated by multiple environmental factors, and the distribution patterns of dominant taxa directly reflect nutrient heterogeneity associated with changes in distance from the hyphosphere of cultivated morels. In the present study, the dominant bacterial phyla across all treatments were Proteobacteria, Acidobacteriota, and Actinobacteriota. Co-occurrence network analysis likewise showed that the bacterial communities were composed predominantly of taxa affiliated with these three phyla, consistent with previous reports [24,25]. At the genus level, *RB41* (affiliated with Acidobacteriota) and *Sphingomonas* (affiliated with Proteobacteria) were the most abundant taxa. Notably, six of the ten dominant genera, including *Dongia* and *Bradyrhizobium*, belonged to Proteobacteria, further confirming the dominant ecological role of this phylum.

Compared with the control, cultivation of *M. sextelata* significantly altered the abundance of specific bacterial phyla in a distance-dependent manner. At 20 cm from the morel growth site (R_20_), the relative abundances of Proteobacteria and Actinobacteriota increased significantly, whereas in the hyphosphere zone immediately adjacent to the morel (R), Acidobacteriota became significantly enriched. These patterns are likely driven by differences in nutrient utilization, gradients of antimicrobial compounds, and microenvironmental heterogeneity.

First, the enrichment of Proteobacteria in R_20_ may be attributed to the copiotrophic and opportunistic nature of this phylum, whose members rapidly exploit soluble organic compounds, such as sugars and organic acids, diffusing from fungal exudates into the surrounding soil [26]. Indeed, R_20_ exhibited the highest total nitrogen, particulate organic carbon (POC), and readily oxidizable carbon (ROC) contents (Supplementary Table S1). In addition, the relative abundance of typical nitrogen-fixing bacteria such as *Bradyrhizobium* reached its peak in this zone. Meanwhile, the nitrogen-cycling genus *Sphingomonas* was also enriched synchronously. These results indicated that R_20_ may be an active region for hypha-associated nitrogen fixation by *M. sextelata* [27,28], thereby stimulating organic nitrogen mineralization [29] and hyphosphere-associated biological nitrogen fixation [12]. Moreover, *Sphingomonas* can secrete carbohydrate-rich compounds into soil [30], which may further promote the proliferation of *Sphingomonas*, *Bradyrhizobium*, and other proteobacterial taxa in R_20_.

The increased abundance of Actinobacteriota in R_20_ may be explained by two complementary mechanisms. On the one hand, because this zone is farther from the dense hyphal core, the concentration of hypha-derived antimicrobial secondary metabolites, such as antibiotics and antimicrobial peptides, is likely reduced by diffusion, thereby alleviating inhibitory effects on actinobacterial populations. On the other hand, secondary metabolites produced by *Sphingomonas* have been linked to the suppression of pathogenic microorganisms [31,32] and degrade toxic compounds [33], potentially improving the local microenvironment indirectly. The high relative abundance of beneficial bacteria in R_20_, particularly *Sphingomonas* and *Bradyrhizobium*, further indicates that this zone may be especially favorable for the selective colonization of functionally important bacteria shaped by *M. sextelata* hyphal exudates, including taxa involved in nitrogen fixation and pathogen suppression.

In contrast, Acidobacteriota dominated in the R zone immediately adjacent to the morel. This area is characterized by dense hyphal aggregation, which may create a micro-oxic environment [34], and it also exhibited the lowest soil pH (Supplementary Table S1). Exudates in the near-hyphal zone are likely enriched in macromolecular compounds such as chitin- and lignin-derived materials [35,36], while organic acids secreted by the hyphae, including oxalic and citric acids, may further acidify the hyphosphere [37]. Because many members of Acidobacteriota are acidophilic, and because their extracellular enzymes, such as cellulases, display higher activity under acidic conditions, these taxa are better adapted to degrade the recalcitrant organic substrates enriched in the R zone, including hyphal residues and lignocellulosic materials. These traits correspond with their highest abundance of Acidobacteriota in this zone, particularly that of *RB41*.

Following morel cultivation, both bacterial diversity and richness indices were highest in the uncultivated control (CK) and lowest in the soil immediately adjacent to the morel hyphae (R), whereas the Dominance index was highest in R. These findings indicate that morel cultivation increased bacterial dominance, resulted in a more uneven species distribution, and reduced overall bacterial diversity. This pattern is consistent with the findings of Shen et al. [38], who reported significantly higher Chao1, Shannon, Simpson, and Pielou indices in non-hyphosphere soil than in hyphosphere soil. This was also consistent with the results of the PLS-SEM analysis, which showed a significant negative correlation between bacterial diversity and dominant bacterial genera (with path coefficients of −0.037). These results suggest that the hyphosphere environment imposes strong resource-based environmental filtering on microbial communities. The specialized substrate derived from *M. sextelata* mycelium selectively enriches well-adapted microbial taxa, which may shape community dominance through multiple pathways, including potential interspecific competition for resources, priority effects of colonizing microorganisms, random community drift, and dispersal limitation along the mycelial distance gradient. This selective screening process gradually eliminates groups that are ill-suited to the specific mycelial layer microhabitat, ultimately leading to a reduction in overall species richness and a decrease in the Chao1 index.

PLS-SEM further showed that the spatial variation in soil chemical properties with hyphosphere distance indirectly and negatively affected dominant bacterial genera (consistent with the negative direct effect of soil chemical properties on dominant bacterial genera, path coefficient = −0.773, *p* < 0.05), while directly and negatively affecting bacterial diversity. Several mechanisms may account for why soil chemical properties negatively drive dominant bacterial genera despite appearing to enhance soil nutrients. First, organic substances secreted by morel hyphae, including polysaccharides and enzymes, together with the addition of exogenous nutrient bags, significantly increased soil nitrogen availability (Supplementary Table S1). However, this nutrient enrichment selectively favors a limited subset of metabolically versatile, copiotrophic bacteria such as Proteobacteria [12]. These taxa efficiently exploit abundant resources and quickly occupy ecological niches, thereby increasing their relative abundance and enhancing community dominance. By contrast, oligotrophic bacteria and taxa dependent on specialized carbon sources are unable to utilize morel-derived carbon substrates efficiently, resulting in suppressed growth and eventual exclusion [38], which leads to a decline in overall bacterial diversity.

Second, morels preferentially grow in slightly acidic environments, and their metabolic activity lowers hyphosphere soil pH (Supplementary Table S1), thereby selecting for acid-tolerant bacteria, such as some members of Acidobacteriota and Alphaproteobacteria [39], while inhibiting neutrophilic or alkalitolerant taxa [40]. Third, morels compete with bacteria for soil nutrients such as ammonium and phosphorus; in this study, total phosphorus decreased after morel cultivation (Supplementary Table S1). Under these conditions, dominant bacterial taxa—especially those engaged in mutualistic interactions with morels—may retain access to nutrients through cooperative metabolism, whereas less competitive bacteria are progressively excluded under nutrient limitation, further compressing community diversity [41]. In addition, morels may secrete allelochemicals, including antimicrobial peptides and phenolic compounds, which inhibit a broad range of bacteria, particularly pathogenic taxa or those occupying overlapping functional niches. In contrast, certain bacteria, including taxa capable of degrading allelochemicals or benefiting from mutualistic interactions with morels, such as *Sphingomonas* and *Bradyrhizobium*, are either unaffected or even promoted. This selective advantage further reinforces community dominance and drives a shift toward a structure controlled by a limited number of highly dominant taxa, ultimately reducing diversity [7]. This likely explains why bacterial diversity and richness were lowest, and the Dominance index highest, in the R zone. Co-occurrence network analysis further supports this interpretation: bacterial network nodes, edges, positive correlations, and stability were all lowest near the hyphae (Region R), indicating that intense hyphal filtering significantly simplified the network structure while reducing diversity and richness.

The PLS-SEM model indicates that enzyme activity negatively regulates dominant bacterial genera (with path coefficients of −0.352) by intensifying nutrient competition and filtering out non-dominant groups; the highest enzyme activity and lowest SOC levels observed in Zone R confirm this mechanism (Supplementary Table S1). In summary, the synergistic effects of physicochemical properties and enzyme activity suppress bacterial diversity while promoting specific dominant genera, thereby validating the hypothesis.

### 4.2. Effects of Under-Forest Morel Cultivation on Fungal Community Composition and Diversity in Hyphosphere Soils at Different Distances

In this study, the dominant fungal phyla in morel hyphosphere soil were Ascomycota and Basidiomycota, and co-occurrence network analysis similarly showed that fungal communities were composed mainly of taxa from these two phyla, in agreement with previous studies by Cui et al. [12] and Yuan et al. [24]. Morel cultivation significantly decreased the relative abundance of Ascomycota, with the greatest decline observed in R_20_, whereas Basidiomycota exhibited the opposite trend, showing the largest increase in R_20_.

The decline in Ascomycota abundance may be explained first by nutrient competition. As a saprotrophic fungus, morel preferentially acquires large amounts of organic matter and available phosphorus in the hyphosphere core zone. Because many ascomycetous fungi occupy ecological niches highly similar to that of morel, they are competitively disadvantaged in adjacent soil, resulting in reduced abundance, whereas competition weakens with increasing distance and their abundance partially recovers [12]. In addition, the rapid extension of morel hyphae occupies soil space and may physically impede colonization by other ascomycetous fungi at close range. A second contributing factor is allelopathic inhibition. Metabolites secreted by morel hyphae, including certain organic acids, may exert concentration-dependent inhibitory effects on many members of Ascomycota [13,41,42], strongly suppressing spore germination and hyphal growth in the immediate vicinity of the mycelium. A third factor may be local environmental unsuitability. Morel growth reduced the hyphosphere soil pH (Supplementary Table S1) and altered enzyme activities, such as decreasing urease activity. Many ascomycetous fungi are more adapted to the conditions of uncultivated soil and may therefore be inhibited in the modified microenvironment generated by morel cultivation [41].

By contrast, the increase in Basidiomycota, especially in R_20_, appears to be mainly related to differences in nutrient use strategies and environmental adaptation [43]. Intermediate metabolites generated during morel hyphal metabolism, including organic acids and sugars, may provide high-quality nutrient sources for basidiomycetous fungi [12,44]. The PLS-SEM results further support these findings, showing that soil chemical properties positively influenced dominant fungal genera (path coefficient = 0.610, *p* < 0.05) and explained 53.9% of the variance in dominant fungal genera. In addition, compared with Ascomycota, Basidiomycota may be better adapted to the modified microenvironment created by morel cultivation, such as lower soil pH [41]. Some basidiomycetous fungi may also maintain cryptic symbiotic or facilitative relationships with morel, using its hyphal network as a physical scaffold for attachment and spread; processes associated with sclerotial formation may likewise create favorable microhabitats for basidiomycete growth. This potential symbiotic or synergistic association—supported by the highest abundance of symbiotrophic fungi in R_20_—may explain why the abundance pattern of Basidiomycota contrasted sharply with that of Ascomycota [45].

Among the dominant genera, *Sebacina* (Basidiomycota) showed its highest abundance at 20 cm from the hyphosphere (R_20_). Mantel analysis revealed positive correlations between *Sebacina* and the activities of carbon- and nitrogen-cycling enzymes, including BG, CBH, and URE. Its hyphal network may therefore enhance nitrogen fixation efficiency, which is consistent with the highest total nitrogen content observed in R_20_ in the present study. In addition, *Sebacina* may provide attachment sites and nutrients for beneficial microorganisms, such as *Sphingomonas* and *Bradyrhizobium*, both of which were most abundant in R_20_, while simultaneously suppressing pathogens and helping maintain microecological balance [46]. These interactions may further explain why Basidiomycota increased most strongly in R_20_ and suggest that this zone may represent the most favorable habitat for beneficial microorganisms associated with *M. sextelata*.

Another dominant taxon was the unclassified Microbotryales_gen_Incertae_sedis (Basidiomycota), which ranked second in abundance and increased following morel cultivation relative to CK. Previous studies suggest that this group may participate in the decomposition of both simple and complex organic substrates, including lignocellulosic materials, thereby promoting nutrient cycling [47,48]. Its elevated abundance may also be associated with strong stress tolerance, suppression of pathogenic fungi, and increased soil organic matter resulting from the addition of exogenous nutrient bags; indeed, R_20_ had the highest organic carbon content and carbon fractions (Supplementary Table S1). However, the specific ecological functions of this taxon in morel hyphosphere soil remain to be clarified.

In the present study, the R zone showed the highest fungal Dominance index but the lowest Shannon, Simpson, and Pielou indices, indicating that morel cultivation increased fungal dominance while reducing fungal diversity. This finding is consistent with the results of Wang et al. [29,49], who also reported that morel cultivation reduced soil fungal diversity. Similarly, the PLS-SEM results revealed a strong negative correlation between fungal diversity and dominant fungal genera, suggesting that the proliferation of dominant taxa actively suppresses community evenness. By contrast, at 40 cm from the hyphosphere (R_40_), the Dominance index was lowest, whereas all other diversity indices were highest, indicating greater species evenness, richness, and overall diversity in this zone.

As the dominant fungal taxon, morel itself proliferates extensively within its active growth zone. On the one hand, it preferentially captures soil carbon, nitrogen, phosphorus, and other nutrients, limiting nutrient availability for other microorganisms and preventing large-scale proliferation of competing taxa. As a consequence, only a few species, such as *Sebacina*, which can secrete extracellular enzymes including cellulases and lignin-degrading enzymes, may gain a marked competitive advantage, leading to a sharp increase in the Dominance index [12]. Moreover, the mycelium of morels secretes extracellular enzymes such as urease (URE) and catalase (CAT) [50], which eliminate reactive oxygen species (such as H_2_O_2_) to mitigate oxidative damage to the fungus and catalyze the hydrolysis of urea to produce ammonia [51], thereby providing the fungus with a directly utilizable inorganic nitrogen source and promoting the proliferation of dominant fungi in the soil. On the other hand, the emergence of such a strongly dominant taxon directly reduces species richness and community evenness, ultimately lowering the Shannon, Simpson, and Pielou indices. Consistent with the PLS-SEM results, enzyme activities (CAT and URE) exert a significant positive regulatory effect on dominant fungal genera (path coefficient = 0.730), further reinforcing the competitive dominance of these taxa.

At 40 cm from the hyphosphere (R_40_), however, the competitive effect of morel for nutrients is substantially weakened, allowing a wider range of microorganisms to access resources more equitably. As a result, species richness and evenness increase, and community dominance correspondingly declines. In addition, morel hyphae may secrete allelopathic or autotoxic compounds such as phenolic acids [52], which inhibit the growth of many non-adapted microorganisms while favoring only a limited subset of tolerant taxa, such as *Sebacina* and the stress-tolerant Microbotryales_gen_Incertae_sedis, thereby further simplifying the fungal community near the hyphae. Because R_40_ is relatively distant from the hyphosphere, it is minimally affected by such allelochemicals, and the strength of environmental filtering is reduced. The lower abundance of morel hyphae in this zone also facilitates invasion and establishment by other fungal taxa, resulting in the highest fungal diversity and evenness indices. Co-occurrence network analysis supported this interpretation: the fungal network in R_40_ had more nodes, edges, average degree, network modulus, and robustness weight, as well as more positive associations, and higher complexity and stability, all of which indicate that reduced selection pressure on fungal hyphae in this area has allowed diverse fungal groups to coexist, restoring the structural resilience and stable modular organization of the soil fungal community and resulting in higher fungal diversity. These findings are consistent with the patterns revealed by species composition and community structure analyses.

## 5. Conclusions

Under-forest cultivation of *M. sextelata* markedly affected the structure and diversity of hyphosphere soil microbial communities in a distance-dependent manner. The dominant bacterial phyla were Proteobacteria, Acidobacteriota, and Actinobacteriota, whereas the dominant fungal phyla were Ascomycota and Basidiomycota. In the R zone immediately adjacent to the hyphosphere, Acidobacteriota predominated. At 20 cm (R_20_), the abundances of Proteobacteria—particularly *Dongia*, *Sphingomonas*, and *Bradyrhizobium*—as well as Actinobacteriota and Basidiomycota (including *Sebacina* and Microbotryales_gen_Incertae_sedis) were highest. At 40 cm (R_40_), fungal diversity reached its maximum. These distance-dependent patterns were primarily driven by microenvironmental changes (soil chemical properties and enzyme activity) shaped by morel hyphal exudates, nutrient competition, and allelochemicals such as phenolic acids. Owing to its relatively abundant carbon and nitrogen resources, R_20_ appeared to provide a favorable habitat for beneficial microorganisms, including nitrogen-fixing and nutrient-cycling taxa. By contrast, the R zone was characterized by an acidic microenvironment, recalcitrant organic substrates, and stronger allelopathic inhibition, resulting in the concentration of a limited number of dominant taxa and the lowest bacterial and fungal diversity. Overall, morel cultivation increased microbial community dominance while reducing total diversity, and its selective filtering effect on soil microorganisms weakened progressively with increasing distance from the hyphosphere.

## Figures and Tables

**Table 1:** Primers and the ITS sequences for microbial diversity determination [23].

Classification	Amplificationregion	Primersequence (5′→3′)
Fungal (ITS) rRNA	ITS1-5F	GGAAGTAAAAGTCGTAACAAGG,GCTGCGTTCTTCATCGATGC
Bacterial 16S rRNA	16SV4	GTGCCAGCMGCCGCGGTAA,GGACTACHVGGGTWTCTAAT

**Table 2:** Characteristics of Alpha diversity indices for bacterial and fungal communities.

Microorganism	Treatments	Chao1	Pielou	Shannon	Dominance	Simpson
bacterial	R	1938.12 ± 116.07 a	0.826 ± 0.006 a	8.970 ± 0.097 a	0.0080 ±0.0007 b	0.9920 ± 0.0007 a
	R_20_	2068.24 ± 151.44 a	0.837 ± 0.009 ab	9.179 ± 0.083 ab	0.0063 ± 0.0010 ab	0.9937 ± 0.0010 ab
	R_40_	2281.52 ± 104.05 a	0.851 ± 0.007 bc	9.464 ± 0.121 bc	0.0060 ± 0.0008 ab	0.9940 ± 0.0008 ab
	CK	2190.72 ± 92.57 a	0.861 ± 0.008 c	9.537 ± 0.111 c	0.0050 ± 0.0007 a	0.9950 ± 0.0007 b
fungi	R	979.34 ± 40.53 a	0.585 ± 0.024 a	5.79 ± 0.24 a	0.074 ± 0.015 b	0.926 ± 0.015 a
	R_20_	946.19 ± 35.61 a	0.635 ± 0.014 b	6.26 ± 0.14 ab	0.048 ± 0.004 a	0.952 ± 0.004 b
	R_40_	1338.78 ± 58.68 c	0.674 ± 0.014 b	6.97 ± 0.19 c	0.030 ± 0.004 a	0.970 ±0.004 b
	CK	1130.57 ± 50.89 b	0.647 ± 0.011 b	6.54 ± 0.15 bc	0.035 ± 0.004 a	0.965 ± 0.004 b

Note: Number of replicates n = 6; different lowercase letters within the same column indicate a statistically significant difference at the 0.05 level.

**Table 3:** Key topological properties of bacterial networks in different treatments.

Network Indices	R	R_20_	R_40_	CK
Nodes	1156	1291	1442	1315
Edges	4360	5680	6105	5619
Distance	6.44	9.37	14.5	3.43
Average degree	7.54	8.80	8.47	8.55
Positive-to-negative edge ratio	51.63	61.50	110.11	54.56
Modularity	0.918	0.868	0.921	0.936
Robustness-weight	0.395	0.389	0.403	0.388

**Table 4:** Key topological properties of fungal networks in different treatments.

Network Indices	R	R_20_	R_40_	CK
Nodes	488	586	729	601
Edges	1041	1674	2087	1383
Distance	4.76	4.45	7.83	4.15
Average degree	4.27	5.71	5.73	4.60
Positive-to-negative edge ratio	249.00	75.92	124.00	99.00
Modularity	0.930	0.843	0.933	0.940
Robustness-weight	0.403	0.402	0.416	0.401

## Data Availability

The original contributions presented in this study are included in the article/Supplementary Materials. Further inquiries can be directed to the corresponding author.

## Supplementary Materials

The following supporting information can be downloaded at: https://www.mdpi.com/article/10.3390/microorganisms14051003/s1, Table S1: The effect fairy ring distance on soil nutrients.
