# Supplementary material for: Spatial Differentiation and Community Structure Characteristics of Soil Microorganisms at Variable Hyphosphere Distances in Forest Cultivation Systems of Morchella
Source: Microorganisms. 2026 Apr 29;14(5):1003. doi: 10.3390/microorganisms14051003 (PMC13209776; doi:10.3390/microorganisms14051003)
Supplement: Supplementary file 1 [file microorganisms-14-01003-s001.zip › microorganisms-4249605-supplementary.pdf]

Table S1. The effect fairy ring distance on soil nutrients.

Soil Physical and Chemical Properties	R	R_{20}	R_{40}	CK
pH	7.226±0.020d	7.312±0.010c	7.371±0.023b	7.544±0.014a
EC (μs·cm^{-1})	178.000±2.309ab	164.667±5.696c	173.333±1.764bc	188.000±2.309a
Total P (mg·g^{-1})	1.853±0.072a	1.723±0.052a	1.817±0.052a	1.917±0.048a
Total N (mg·g^{-1})	4.389±0.171a	4.417±0.208a	4.236±0.141a	4.031±0.048a
SOC (mg·g^{-1})	46.177±4.397c	68.557±2.291a	57.150±0.752b	64.080±3.882ab
ROC (mg·g$^{-1)}$	0.617±0.018c	0.960±0.010a	0.610±0.015c	0.737±0.026b
POC (mg·g^{-1})	0.783±0.097	0.943±0.043	0.747±0.032	0.767±0.027
MOC (mg·g^{-1})	45.393±4.323c	67.613±2.250a	56.403±0.752b	63.313±3.905ab
CAT (ml·g^{-1})	11.527±0.077b	12.027±0.116a	12.293±0.096a	12.080±0.023a
CBH (nmol·g^{-1}·h^{-1})	32.258±0.938a	21.508±0.467c	31.222±0.618ab	29.865±0.326b
BG (nmol·g^{-1}·h^{-1})	61.143±0.426a	52.220±0.438c	59.955±0.911ab	58.128±0.664b
URE (mg·d^{-1}·g^{-1})	0.830±0.010c	1.17±0.015b	1.310±0.010a	1.153±0.019b

R, Soil zone 0 cm from Morchella growth site; R_{20}, Soil zone 20 cm from Morchella growth site; R_{40}, Soil zone 40 cm from Morchella growth site. CK, Soil zone without Morchella cultivation. Different lowercase letters in the same column indicate significant differences among rhizosphere distances ($P< 0.05$).
